# Endonuclease domain of the *Drosophila melanogaster* R2 non-LTR retrotransposon and related retroelements: a new model for transposition

**DOI:** 10.3389/fgene.2013.00063

**Published:** 2013-04-26

**Authors:** Dmitry V. Mukha, Elena G. Pasyukova, Tatiana V. Kapelinskaya, Arina S. Kagramanova

**Affiliations:** ^1^Vavilov Institute of General Genetics, Russian Academy of SciencesMoscow, Russia; ^2^Institute of Molecular Genetics, Russian Academy of SciencesMoscow, Russia

**Keywords:** non-LTR retrotransposons, target-specific retrotransposition, endonucleases, Holliday junction-resolving enzymes, R-loops

## Abstract

The molecular mechanisms of the transposition of non-long terminal repeat (non-LTR) retrotransposons are not well understood; the key questions of how the 3′-ends of cDNA copies integrate and how site-specific integration occurs remain unresolved. Integration depends on properties of the endonuclease (EN) domain of retrotransposons. Using the EN domain of the *Drosophila* R2 retrotransposon as a model for other, closely related non-LTR retrotransposons, we investigated the EN domain and found that it resembles archaeal Holliday-junction resolvases. We suggest that these non-LTR retrotransposons are co-transcribed with the host transcript. Combined with the proposed resolvase activity of the EN domain, this model yields a novel mechanism for site-specific retrotransposition within this class of retrotransposons, with resolution proceeding via a Holliday junction intermediate.

## Introduction

Eukaryotic transposable elements (TEs) are ubiquitous components of eukaryotic genomes that are important for shaping the genetic material. New copies of TEs integrate into new sites in the genome and can cause genomic and genetic variations. New insertions can: (1) alter gene expression by providing *cis*-regulatory elements, such as promoters, enhancers, and transcription factor binding sites; (2) induce insertion-mediated deletions; or (3) affect chromosome replication, recombination, and pairing. The spread of regulatory elements by TEs can lead to the creation of specific regulatory networks, induce pathologies including cancer, affect host environmental adaptations, or contribute to genetic diversity. TEs thus have a large impact on genome evolution (for review, see Oliver and Greene, 2009; Bire and Rouleux-Bonnin, 2012; Kim et al., 2012; Casacuberta and González, 2013; Chénais, 2013). Therefore, understanding the mechanisms of TE dissemination, in particular, the mechanism of transposition, is of great general importance.

Eukaryotic TEs can be divided into two types: retrotransposons and DNA transposons. All retrotransposons are transposed through an RNA intermediate. Messenger RNA from the retrotransposons is expressed in host cells, and after reverse transcription by reverse transcriptases (RTs) that are encoded by the TEs, new DNA copies of the elements are integrated into new sites within the host genome. In contrast, DNA transposons are transposed from one genome site to another by the movement of DNA copies through the activity of DNA transposases encoded by the TEs (Craig et al., 2002; Kapitonov et al., 2009; Oliver and Greene, 2009; Bire and Rouleux-Bonnin, 2012).

Four classes of retrotransposons are present in the genomes of all eukaryotes: non-long terminal repeat (non-LTR), LTR, Penelope, and DIRS retrotransposons (Craig et al., 2002; Evgen'ev and Arkhipova, 2005; Poulter and Goodwin, 2005). Based on structural features and RT domain phylogeny, non-LTR retrotransposons are divided into five main groups: R2, L1, RTE, I, and Jockey; these are subdivided into 28 clades (Kapitonov et al., 2009). The R2 group includes the most ancient clades: CRE, NeSL, R2, Hero, and R4. Members of these clades are characterized by a single open reading frame (ORF) for the RT domain followed by an endonuclease (EN) domain that is similar to PD-(D/E)XK nucleases (Burke et al., 1999; Yang et al., 1999; Kojima and Fujiwara, 2005a). Members of the L1, RTE, I, and Jockey groups encode an apurinic-apyrimidinic EN (APE), adjacent to the RT domain at the N-terminal end (Zingler et al., 2005). A new Dualen (also called Rand I) group of non-LTR retrotransposons with unusual properties has been described recently (Kojima and Fujiwara, 2005b). These retrotransposons code for both an APE EN and an EN similar to PD-(D/E)XK nucleases.

PD-(D/E)XK nucleases (named for the highly conserved active site motif) are extremely diverse, and involved in nucleic acid metabolism: DNA restriction (Roberts et al., 2003), bacteriophage λ recombination (Kovall and Matthews, 1997), DNA damage repair (Ban and Yang, 1998; Tsutakawa et al., 1999), Holliday junction resolution (Hadden et al., 2001; Nishino et al., 2001, 2003; Middleton et al., 2003), and RNA processing (Dias et al., 2009; Xiang et al., 2009; Yuan et al., 2009). Although PD-(D/E)XK domains often have little sequence similarity overall, they share a structurally conserved core of a four-stranded mixed β-sheet flanked by an α-helix on each side (αβββαβ topology) (Feder and Bujnicki, 2005; Kinch et al., 2005).

The mechanisms by which R2 group retrotransposons integrate into host genomes has been analyzed *in vitro* (Bibillo and Eickbush, 2002, 2004; Christensen and Eickbush, 2005; Christensen et al., 2005, 2006; Kurzynska-Kokorniak et al., 2007) and *in vivo* (Eickbush et al., 2000; Eickbush and Eickbush, 2003; Fujimoto et al., 2004) using the R2 retrotransposon of *Bombyx mori*. In addition, sequences from 12 *Drosophila* genome projects were analyzed to address questions on the evolution and mechanism of R2 non-LTR retrotransposon integration (Stage and Eickbush, 2009). R2 EN first nicks one strand of the chromosomal target site. The 3′-hydroxyl group released by this nick is used as the primer for the R2 RT for cDNA synthesis (Luan et al., 1993; Luan and Eickbush, 1995). This mechanism, called target-primed reverse transcription (TPRT), is believed to be the integration mechanism of other non-LTR retrotransposons (Craig et al., 2002) and mobile bacterial and mitochondrial group II introns (Zimmerly et al., 1995). However, the mechanisms of top-strand cleavage and second-strand synthesis are debated; no common mechanisms have been observed. For example, *in vitro* results support a mechanism in which the second strand of the R2 DNA is synthesized by the R2 RT after it exchanges the retrotransposon RNA template for the cDNA template (Kurzynska-Kokorniak et al., 2007). *In vivo*, recombination during formation of the 5′-end of the R2 DNA has been demonstrated (Fujimoto et al., 2004). In *Drosophila*, the 5′-ends of the R2 RNA transcripts are proposed to contain terminal G residues that, after reverse transcription and top-strand cleavage, enable annealing of terminal C residues to G residues in the top DNA strand after cleavage. Cleavage of the top strand by *Drosophila* R2 EN is thought to not be rigidly determined (Stage and Eickbush, 2009). Thus, the means by which the 3′-ends of cDNA copies integrate remain unknown. Despite similarities, transposition mechanisms for different types of non-LTR retrotransposons probably differ in their details.

Another unresolved are the mechanisms that ensure site-specificity of non-LTR retrotransposon integration. From our point of view, in addition to interaction specificity between retrotransposon proteins and target DNA, other mechanisms must ensure non-random selection of integration sites.

In this study, the R2 retrotransposon of *Drosophila melanogaster* was used as a model for the structural and functional features of EN domains of R2 group non-LTR retrotransposons. Analysis of the EN domain protein structure with a canonical αβββαβ topology allowed determination of the EN cleavage domain boundaries. This domain had significant structural similarity with Holliday junction-resolving enzymes from Archaea. Based on these and previous findings from other studies, we propose a new model of transposition explaining the possible mechanism of top strand cleavage and site-specific integration. In this model, target-specific R2-related retrotransposons that are actively transcribed with their target sequence transpose through the formation of Holliday junction structures. We propose a principle scheme for this new model for a particular type of non-LTR retrotransposons.

## Materials and methods

The general domain architecture of proteins encoded by ORFs of the retrotransposons was analyzed using the Simple Modular Architecture Research Tool (Letunic et al., 2012) (http://smart.embl-heidelberg.de/smart/set_mode.cgi?NORMAL=1). Homology detections, HMM-HMM comparisons, and protein three-dimensional (3D) structure predictions used Protein Homology/analogY Recognition Engine V2.0 (PHYRE-2) (Kelley and Sternberg, 2009) (http://www.sbg.bio.ic.ac.uk/phyre2/html/page.cgi?id=index) and HHpred (Söding, 2005; Söding et al., 2005) (http://toolkit.tuebingen.mpg.de/hhpred#). Pairwise comparison of protein structures used the DaliLite-pairwise option (version 3.1) (Hasegawa and Holm, 2009) (http://ekhidna.biocenter.helsinki.fi/dali_lite/start). To evaluate the quality of 3D protein structure predictions we used ProQ (Wallner and Elofsson, 2003) (http://www.sbc.su.se/~bjornw/ProQ/ProQ.cgi). For global alignment of compared amino acid sequences, we used Basic GeneBee ClustalW 1.83 (http://www.genebee.msu.su/clustal/). For comparative protein structure modeling by satisfaction of spatial restraints followed by estimation of model quality we used Modeller (Sali et al., 1995) and Verify3D (Luethy et al., 1992) (http://toolkit.tuebingen.mpg.de/modeller).

## Results and discussion

### Endonuclease cleavage domain boundaries in the *D. melanogaster* R2 non-LTR retrotransposon ORF

The structural and functional organization of the non-LTR retrotransposons related to the R2 group has been reported in numerous studies. Based on sequence comparisons and biochemical experiments, the EN domain of the R2 retrotransposons was suggested to be similar to *Fok*I-like restriction enzymes (Burke et al., 1999; Yang et al., 1999). *Fok*I is in an unusual class of restriction enzymes that recognize a specific DNA sequence and cleave a short distance away. *Fok*I has an N-terminal DNA recognition domain and a C-terminal cleavage domain (Wah et al., 1998). Similarly, the ORF encoded by the R2-like retrotransposons have a DNA-binding motif (CCHC) and a potential EN cleavage domain. Using new, highly sensitive methods for protein similarity detection and structure prediction such as HMM-HMM-comparison and the large number of new crystal structures of PD-(D/E)XK nucleases, we performed a new search for domains homologous to the EN cleavage domain of the R2 retrotransposons, to predict their 3D structure.

Our first goal was to identify the boundaries of the EN cleavage domain within the *D. melanogaster* R2 retrotransposon ORF, before further similarity searches and functional predictions. We assumed that the boundaries of the EN cleavage domain would be defined by the ends of the canonical structure with the αβββαβ topology typical of PD-(D/E)XK nucleases.

**Figure 1:**
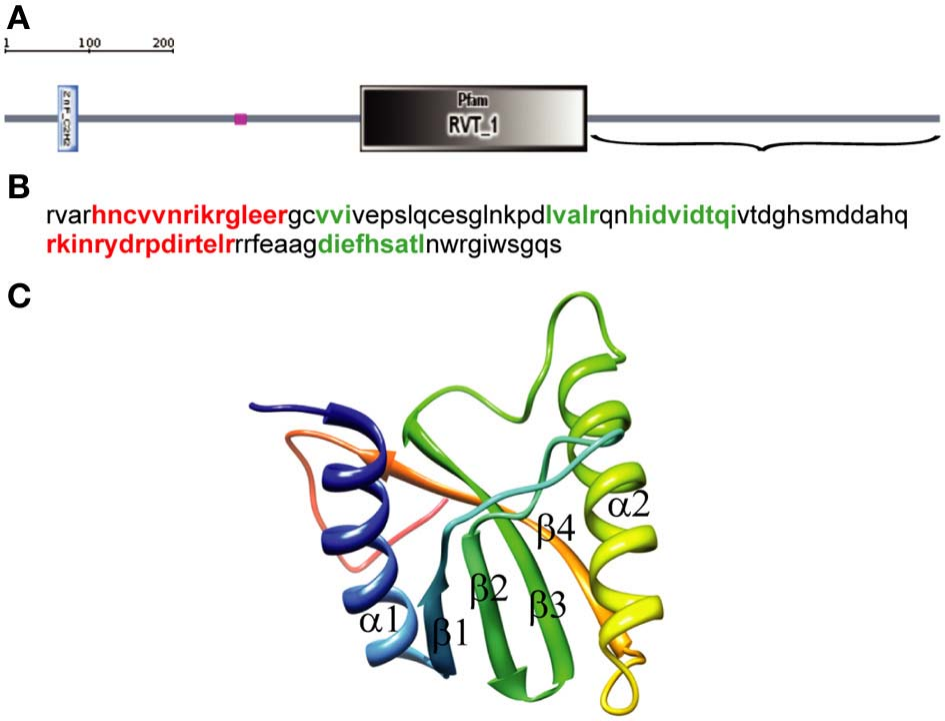
**Identification of the endonuclease domain in the ORF of the *Drosophila melanogaster* R2 non-LTR retrotransposon. (A)** Domain architecture using simple modular architecture research tool (SMART). The three structural elements are blue, zinc-finger domain (aa 61–84); purple, region of low compositional complexity (aa 261–274); gray, RT domain (aa 403–660). Parentheses, C-terminal end of the R2 retrotransposon ORF sequence (aa 661–1057) used to identify the EN domain boundaries. **(B)** Sequence for αβββαβ topology. Red, α-helices; green, β-sheets. **(C)** 3D structure of the 109-aa sequence predicted by PHYRE-2, α-helixes and β-sheets.

We used SMART with default parameters to analyze 1057 amino acids (aa) of the *D. melanogaster* R2 retrotransposon ORF. Three structural elements were identified: a zinc-finger domain (aa 61–84), a region of low compositional complexity (aa 261–274), and an RT domain (aa 403–660) (Figure 1A). The EN domain should be downstream of the RT domain, so only the C-terminal end of the ORF sequence (aa 661–1057) (Figure 1A), was further analyzed. The 3D structure of this 397-aa sequence was predicted using PHYRE-2 in intensive modeling mode. A canonical EN structure with the αβββαβ topology was predicted in the analyzed sequence (data not shown). A 109-aa sequence, including the 95-aa αβββαβ fragment flanked by short sequences marking its boundaries (Figure 1B), was analyzed further. The 3D structure predicted using PHYRE-2 for the 109-aa sequence confirmed the canonical αβββαβ structure (Figure 1C). In the absence of the flanking sequences, PHYRE-2 failed to yield the αβββαβ structure. Based on these results, we concluded that the 95-aa sequence with αβββαβ topology between aa 909 and 1003 of the R2 ORF corresponds to the minimum EN cleavage domain of the *D. melanogaster* R2 retrotransposon. The 109-aa sequence was used for further analyses.

### Similarity detection and structure prediction of the *D. melanogaster* non-LTR R2 retrotransposon EN domain

For similarity detection and structure prediction by HMM–HMM comparison we applied two types of software with default parameters. HHpred was used for initial detection of HHsearch PDB hits, and Phyre-2 was used for more accurate structure comparisons. Finally, global pairwise sequence alignment followed by comparative protein structure modeling by satisfaction of spatial restraints were used for full-length protein structure comparisons.

**Figure 2:**
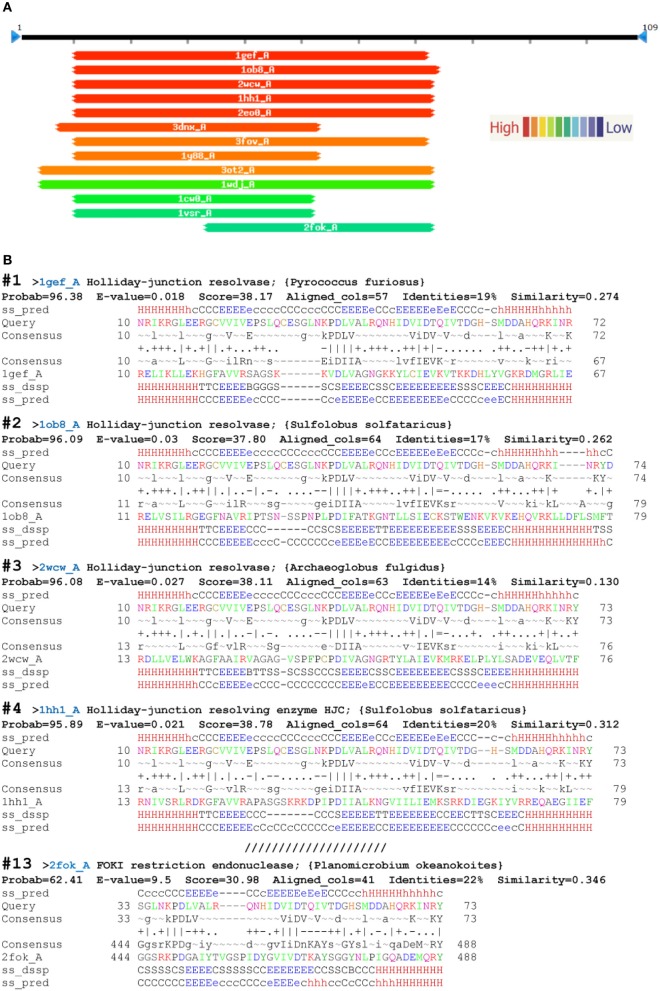
**Top HHsearch PDB hits from a query with the endonuclease domain of the *Drosophila melanogaster* R2 non-LTR retrotransposon. (A)** Hitlist graphical representation; **(B)** HHpred alignments (PDB accessions numbers in parentheses): 1—Holliday-junction resolvase of *Pyrococcus furiosus* (1gef); 2—Holliday-junction resolvase of *Sulfolobus solfataricus* (1ob8); 3—Holliday-junction resolvase of *Archaeoglobus fulgidus* (2wcw); 4—Holliday-junction resolving enzyme of *S. solfataricus* (1hh1); 13—FOKI restriction endonuclease of *Planomicrobium okeanokoites* (2fok). ss_pred, secondary-structure prediction by PSIPRED (H, α-helix; E, β-sheet; C, coil, absence of regular secondary structure); ss_conf, PSIPRED confidence values (0–9); consensus, query alignment consensus sequence, with uppercase >60% and lower case >40% probability. Column scores: =, below −1.5; −, −1.5 to −0.5;., −0.5 to +0.5; +, +0.5 to +1.5; |, above +1.5. The first and last amino acids of the compared sequences are indicated.

The first five PDB hits using HHsearch were archaeal Holliday-junction resolving enzymes with known structures (PDB acc. No: 1gef_A, 1ob8_A, 2wcw_A, 1hh1_A, 2eo0_A). Figure 2 shows four of the five top structural alignments. Substantial similarity was found within the 62- to 64-aa regions with the first α-helix, the following three β-sheets, and the second α-helix (α1β1β2β3α2) of the αβββαβ topology of *D. melanogaster* EN cleavage domain (Figure 2). The E-values for similarity between the query sequence and each of the top Holliday-junction resolving enzymes were significant and well below the threshold level of 1 (Söding, 2005; Söding et al., 2005). *Fok*I was 13 in the PDB hits, with a lower level of similarity to the query sequence than other hits. Substantial similarity between the query sequence and *Fok*I was found only within the 40-aa regions of β2β3α2 of the αβββαβ topology of the studied EN domain (Figure 2). The E-value for the similarity between the query sequence and *Fok*I was 9.5 (much higher than 1), which was not significant. Of note, for all that, it was experimentally shown that R2 EN domain possess the restriction enzyme activity (Luan et al., 1993; Luan and Eickbush, 1995).

**Figure 3:**
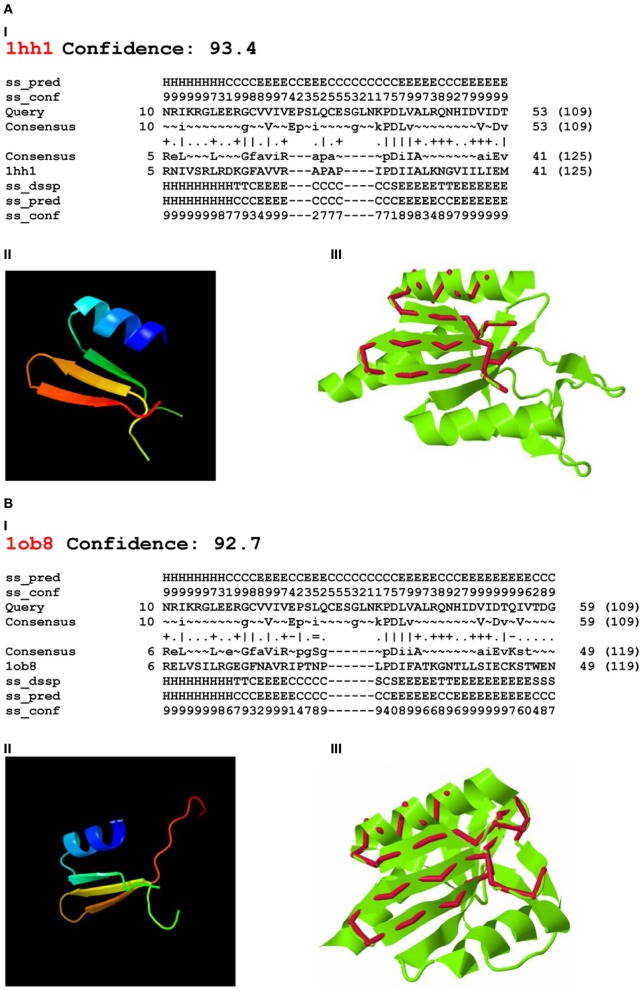
**Phyre-2 comparisons of the endonuclease domain of the *Drosophila melanogaster* R2 non-LTR retrotransposon with HHpred Hits.** Hits: **(A)** Holliday-junction resolving enzyme of *Sulfolobus solfataricus* (1hh1); **(B)** Holliday-junction resolvase of *S. solfataricus* (1ob8). **I**—secondary structure prediction, abbreviations as in Figure 2; **II**—3D structure of the queried sequence based on HHpred alignment; **III**—comparison of 3D structures. Red, queried sequence; green, 3D structure (PDB). Confidence, probability (from 0 to 100) that a match between the query sequence and a given template is a true homology, with >90% confidence that the query protein adopts the overall fold shown and the core is modeled at high accuracy (2–4 Å rmsd from native, true structure).

Using Phyre-2 the first two PDB hits with known function were archaeal Holliday junction resolving enzymes: Holliday junction cleavage (Hjc, confidence level 93.4) and Holliday junction EN (Hje, confidence level 92.7) from *Sulfolobus solfataricus* (PDB acc. No. 1hh1 and No. 1ob8, Figure 3). The confidence levels over 90% indicated that the query protein adopted the overall fold predicted and that the core of the protein was modeled at high accuracy (Kelley and Sternberg, 2009).

Four-way DNA (Holliday) junction-resolving enzymes have been isolated from many organisms, including bacteria and their phages, yeasts and archaea, and mammalian cells and viruses (for review, see Lilley and White, 2001; West, 2003; Lilley, 2010). Archaeal Hjc (1hh1) and Hje (1ob8) are relatively short: 143 and 135 aa, respectively. Despite sharing approximately 30% sequence identity, they cut different strands of the same four-way junction, at different distances from the center (Middleton et al., 2004; White, 2011).

The 3D structures of fragments of the EN domain of the *D. melanogaster* R2 retrotransposon built from the Hjc and Hje templates and corresponding to the structural alignments in Figures 3A,**I** and B,**I** are in Figures 3A,**II** and B,**II**. The quality of the 3D structures was tested by ProQ. For the first model (Figure 3A,**II**) the predicted LGscore was 2.331 and MaxSub—0.377; for the second model (Figure 3B,**II**) the predicted LGscore was 1.858 and MaxSub—0.299. These values indicated that both models were fairly good (LGscore >1.5; MaxSub >0.1).

Comparison of the predicted 3D structures of the *D. melanogaster* R2 EN domain (Figures 3A,**II** and B,**II**) with the experimentally defined 3D structures of Hjc and Hje showed that the predicted α1β1β2β3 of the EN domain perfectly aligned with the α1β1β2β3 of both Holliday junction resolving enzymes (Figures 3A,**III** and B,**III**). Of note, the region with close structural alignment of the EN domain to Hjc and Hje corresponded to the nuclease domains that are responsible for four-way Holliday junction cleavage (Kvaratskhelia et al., 2000).

**Figure 4:**
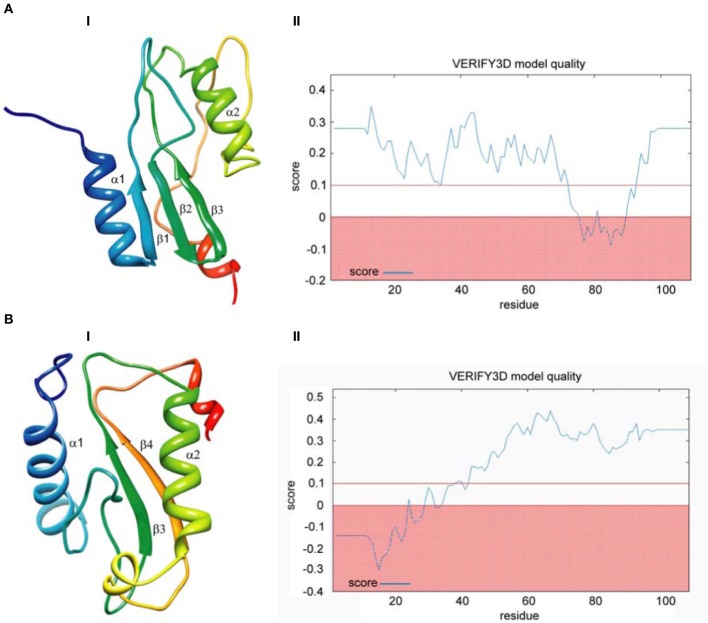
**Comparative protein structure modeling by satisfaction of spatial restraints based on global pairwise sequence alignment of the endonuclease cleavage domain sequence of the *Drosophila melanogaster* R2 non-LTR retrotransposon. (A)** Holliday-junction resolving enzyme of *Sulfolobus solfataricus* (1hh1); **(B)** *Fok* I restriction endonuclease of *Planomicrobium okeanokoites* (2fok). **I**—3D structure prediction; **II**—Verify3D analysis of compatibility of atomic models (3D) with their own amino acid sequences.

**Figure A1:**
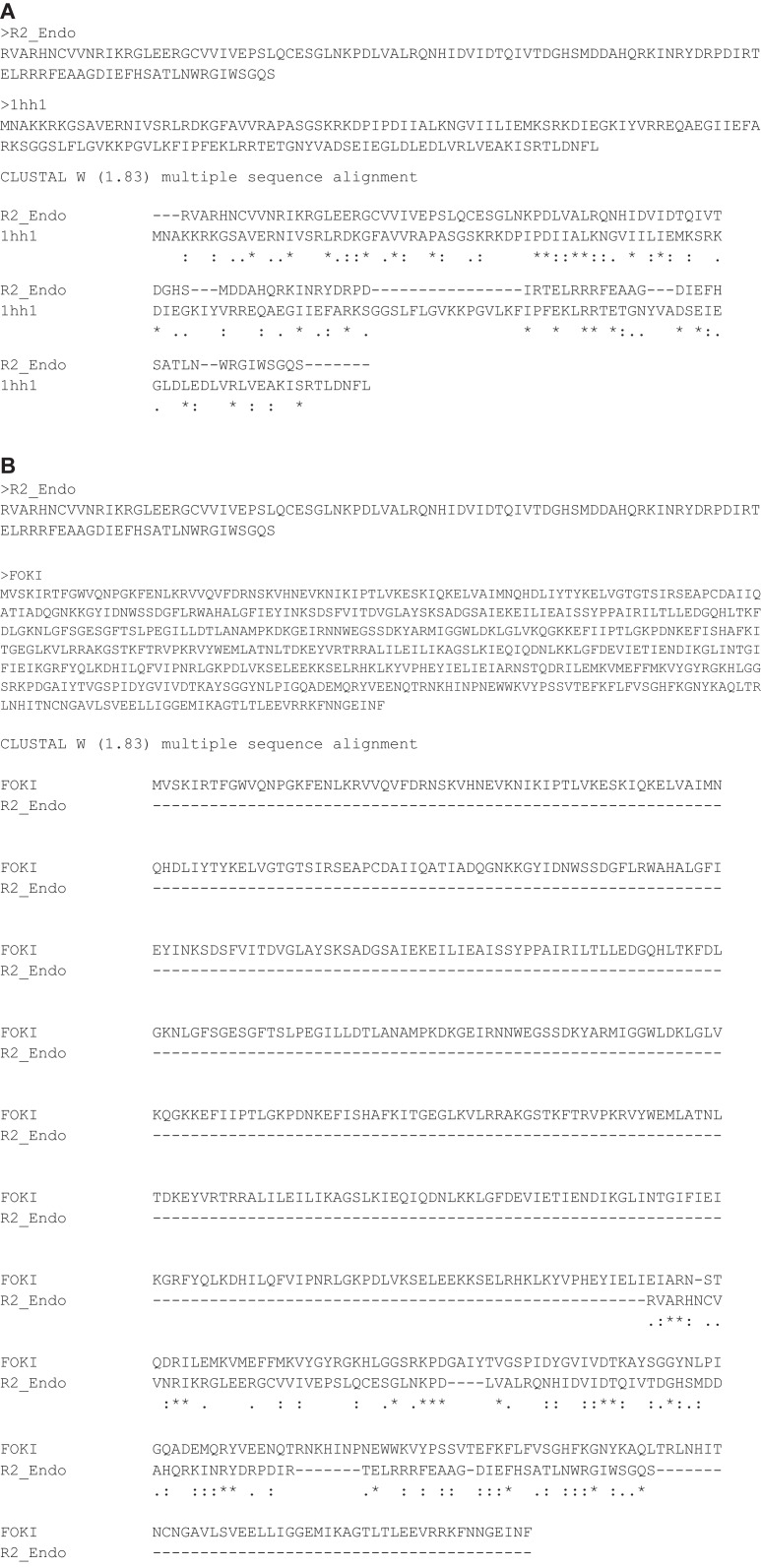
**Global alignment of the *D. melanogaster* R2 EN cleavage domain (R2_Endo) and (A) archaeal Holliday-junction resolvase (1hh1) and (B) restriction endonuclease *Fok*I (FOKI).** The amino acid sequence of the *D. melanogaster* R2 EN cleavage domain aligns with the *Fok*I restriction endonuclease C-terminal cleavage domain (Wah et al., 1998).

Finally, we built models based on global alignments of the *D. melanogaster* R2 EN cleavage domain and the archaeal Holliday junction resolvase (1hh1) and restriction EN *Fok*I (Figure A1). The results of the comparative protein structure modeling by satisfaction of spatial restraints and appropriate graphs evaluating model quality are in Figure 4. Generally, the global alignments results were similar to results from the HMM–HMM comparison. Global alignment of the *D. melanogaster* R2 EN cleavage domain and the archaeal Holliday junction resolvase (1hh1) showed a maximum structural similarity within the α1β1β2β3 structures that are responsible for four-way Holliday junction cleavage (Kvaratskhelia et al., 2000) (Figure 4A,**I**). The result of Verify3D analysis of the compatibility of a 3D atomic model with its aa sequence showed a reliable level of similarity within the aa sequence of the α1β1β2β3 structures (Figure 4A,**II**). Global alignment of the *D. melanogaster* R2 EN cleavage domain and restriction EN *Fok*I (2fok) showed maximum structural similarity within the β3α2β4 structures (Figure 4B,**I**) and Verify3D analysis verified this result (Figure 4B,**II**).

### Integration sites for R2 group non-LTR retrotransposons

Retrotransposons of the R2 group include the clades R2, R4, CRE, NeSL, and Hero. We assumed that phylogenetic closeness of mobile elements in the R2 group, as identified by comparing their RT domains, might reflect the sequence and substrate preference similarity of their EN cleavage domains.

Most R2 group retrotransposons are site-specific and integrate into actively transcribed regions of the genome. R2, R4, and R5 retrotransposons insert in a site-specific manner into 28S rDNA, except for R2 retrotransposons of *Schistosoma*, which insert into the rDNA intergenic spacer (Burke et al., 1995, 2003; DeMarco et al., 2005). R8 retrotransposon of *Hydra magnipapillata* inserts into 18S rDNA (Kojima et al., 2006). CRE retrotransposons were described initially in *Trypanosoma* and *Crithidia* (Aksoy et al., 1990; Gabriel et al., 1990; Villanueva et al., 1991), whereas NeSL retrotransposons were found and described in detail in *Caenorhabditis* (Malik and Eickbush, 2000). Most previously described CRE and NeSL retroelements insert into specific sequences termed miniexons, or spliced leaders. Miniexons are important in the transsplicing required for the correct translation of all or almost all *Trypanosoma* and *Crithidia* species RNAs (Lasda and Blumenthal, 2011). The mRNAs of some *Caenorhabditis* species can also undergo transsplicing (Lasda and Blumenthal, 2011; Morton and Blumenthal, 2011).

The 18S/28S rDNA and the intergenic spacer of rDNA are transcribed by RNA polymerase I (Mayer et al., 2006; Albert et al., 2012), whereas spliced leader sequences are transcribed by RNA polymerase II (Lasda and Blumenthal, 2011; Morton and Blumenthal, 2011). Insertion of TEs into these sequences does not necessarily terminate their transcription. Moreover, we assumed that integrated copies of retrotransposons are transcribed with the target sequences. For *D. melanogaster* (Ye and Eickbush, 2006; Eickbush and Eickbush, 2010) and *Blattella germanica* (Kapelinskaya et al. unpublished data), it was experimentally shown that R2 retrotransposons are transcribed together with 28S rRNA. Unfortunately, we know of no experimental data on the transcription of CRE and NeSL retroelements. We propose that transcription of retrotransposons with target sequences could be important for understanding the mechanisms of integration of these mobile elements.

Some non-LTR retrotransposons obviously in the R2 group (for example, EhRLE3, HEROFr, HEROTn, HERODr, and YURECi) are not inserted into specific target sites and others (for example, DongAg and DongBg) are inserted into microsatellite repeats (Kojima and Fujiwara, 2004). Moreover, some non-LTR retrotransposons in Repbase (the database of repetitive DNA elements, http://www.girinst.org/repbase/index.html) clearly belong to the R2 group but do not seem to be target specific. These non-LTR retrotransposons were mainly detected by computational methods for genome-wide identification of mobile genetic elements. Most retrotransposons in the R2 group do not have their own promoters (Craig et al., 2002; Eickbush and Eickbush, 2010). Thus, if a copy of a mobile element is integrated into the non-transcribed portion of the genome and does not have its own promoter, it is a “dead” copy of a mobile element that has emerged in this part of the genome from non-homologous recombination or the activity of a putative “master copy” of the mobile element.

To add to the complexity, mobile elements (named MoTeR) in a new class of telomere-targeted retrotransposons unique to fungi were recently described (Starnes et al., 2012). MoTeR retroelements are related to the CRE clade retroelements (Starnes et al., 2012), that is, the R2 group. However, based on the structural organization of MoTeR retroelements and their integration sites (telomeric repeats), a unique scheme has been proposed for transposition of these mobile elements (Starnes et al., 2012). This mechanism is significantly different from previous models of R2 transposition (Fujimoto et al., 2004; Eickbush and Jamburuthugoda, 2008; Stage and Eickbush, 2009; Han, 2010).

The bewildering range of integration sites for this class of retrotransposons may suggest a variety of retrotransposition mechanisms. However, we propose a new model for R2-related retrotransposons, characterized by integration sites within actively transcribed regions of the genome, that remains applicable across this diversity.

### A new model for the transposition of R2-related retrotransposons

The structural similarity between the EN domain of the R2 retrotransposon of *D. melanogaster*, the archaeal Holliday junction resolvases, and the restriction EN *Fok*I led us to consider that the EN domain might have activities characteristic of both resolvases and restrictases. We hypothesized that retrotransposon transposition might occur via formation and resolution of Holliday structures. The first nick that starts the TPRT might result from the restriction EN activity of the EN domain. The second nick might occur after Holliday junction formation and occur through the Holliday junction-resolving activity of the EN domain.

A fundamental difference between R2-group retrotransposons is related directly to their transposition mechanism, specifically changes in the target site structure after integration. Target site duplications are formed during the retrotransposition of all known CRE and R4 retroelements and a small number of retrotransposons related to the R2 clade, for example, R8 from *H. magnipapillata*, and R9 from *Adineta vaga* (Burke et al., 1995; Kojima et al., 2006; Gladyshev and Arkhipova, 2009). In contrast, target site deletions are associated with the transposition of all described NeSL retrotransposons and the majority of retrotransposons related to the R2 clade. A few retrotransposons insert into new locations without target site alteration, for example, R2 from *D. melanogaster* and *Nasonia vitripennis* (Kojima et al., 2006). Only the cleavage site of R2 from *B. mori* has been characterized experimentally (Luan et al., 1993). In general, the choice between duplication and deletion of the target site during the course of transposition is thought to depend on the location of the second DNA nick with respect to the first nick. Duplication of the target site occurs if the EN makes the second nick downstream of the first nick. Deletion occurs if the second nick is upstream of the first. No changes occur within the target site after blunt cuts. To account for this, we propose several schemes with minor differences.

Our model is based on several findings and assumptions. (1) The R2 protein could bind both the 3′- and 5′-ends of the template RNA; thus it is likely that a complex that contains one RNA molecule and two R2 protein molecules interacts with the target site on the chromosome (Christensen et al., 2006). (2) The R2 protein can displace RNA or DNA annealed to a DNA template (Kurzynska-Kokorniak et al., 2007). (3) R2 reverse transcriptase can efficiently use single-stranded DNA (ssDNA) as a template (Kurzynska-Kokorniak et al., 2007). (4) R2 EN can cleave ssDNA that extends from either end of a double-stranded region (Kurzynska-Kokorniak et al., 2007). (5) When R2 RNA is added to the R2 protein, a protein homodimer is formed, which enables cleavage of both the upper and lower DNA strands (Yang and Eickbush, 1998). (6) Similarly to other resolvases, both Hjc and Hje are homodimers with two identical active sites that have the same core structure (Middleton et al., 2004). (7) Long non-coding RNAs play an important role in maintenance of the nuclear architecture and in the regulation of gene expression due to sequence complementarity, which enables the formation of RNA–DNA duplexes (R-loops) (Aguilera and García-Muse, 2012; Rinn and Chang, 2012). (We suppose that long RNAs that correspond to the mobile elements and their flanking sequences can interact with target genomic DNA and form R-loop structures). To date, the formation of R-loops in areas of integration of retrotransposons has not been confirmed experimentally. Similarly, there are no data to support the assumption that proteins of retrotransposons could contribute to melting of the DNA strands and, consequently, to the formation of R-loops. At the same time, it is known that the formation of R-loops plays an important role in the maintenance of the structural and functional organization of eukaryotic genomes, and multiple genome sites involved in the formation of these structures have been identified (Wongsurawat et al., 2011). Moreover, it was shown that a single stranded DNA nick can serve as a strong R-loop initiation site (Roy et al., 2010). (8) The R2 EN domain and Holliday junction-resolving enzymes have similar structures (this paper). (9) R2 retrotransposons are integrated into actively transcribed sites within the host genome and are transcribed together with surrounding sequences (Ye and Eickbush, 2006; Eickbush and Eickbush, 2010); it is most likely that the same is true for the most part of TEs that are related closely to the R2 retrotransposon (this paper).

**Figure 5:**
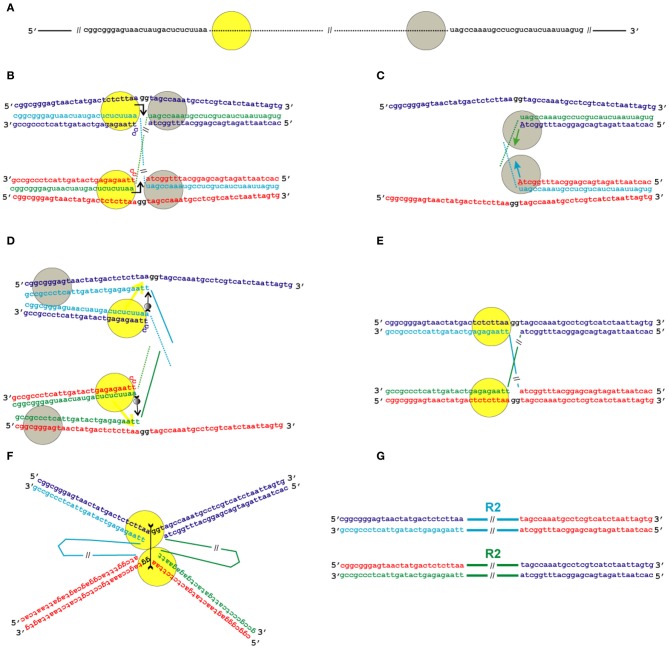
**Model for the transposition of the R2 group of non-LTR retrotransposons for which the endonuclease makes the second DNA nick upstream of the first one.** Sequences correspond to target sites of the *Bombyx mori* R2 non-LTR retrotransposon localized within the 28S rDNA. **(A)** Schematic presentation of two retrotransposon proteins (yellow and gray circles) bound to the RNA co-transcript that contains both the retrotransposon sequence (dotted line) and sequences that flank the target site. **(B)** Proteins, while bound to the RNAs, bind DNA target sites that are located on the homologous chromosomes. The proteins contribute to the melting of the DNA strands, and RNA/DNA duplexes (R-loops) are formed. The protein bound close to the 5′-end of the RNA (yellow circle) makes the first ssDNA nick (depicted by a bent arrow). Two nucleotides marked in black (gg) will be deleted during integration of the mobile element. **(C)** Target primed reverse transcription is initiated by the protein bound close to the 3′-end of the RNA (gray circles). The nucleotides that are donors of the hydroxyl group are depicted in upper-case letters. **(D)** When synthesis of the retrotransposon cDNA (solid line) is complete, the protein (gray circles) rests against the RNA/DNA hybrid. Subsequently, this protein jumps to the corresponding free single DNA strand (the jump is depicted by an arrow with a small gray circle) and continues synthesis of the complementary DNA strand. The protein marked as a yellow circle moves (yellow arrow) from the end of the RNA/DNA hybrid to the end of the newly synthesized dsDNA. Most probably, the RNA in the RNA/DNA hybrids is digested by endogenous host RNase H. **(E)** As a result of the processes described above, a typical Holliday junction structure is formed, with two proteins (yellow circles) bound to the target sites that are located in the two homologous chromosomes. **(F)** Two proteins form the dimer that shows Holliday junction-resolving activity. The second cut is shown by an arrow with two feathers. **(G)** The copies of the retrotransposon that are integrated into the homologous chromosomes are shown. The host DNA polymerase completes the synthesis of the second DNA strand that corresponds to the mobile element and, owing to 5′-3′ exonuclease activity, removes the non-complementary nucleotides (gg).

**Figure 6:**
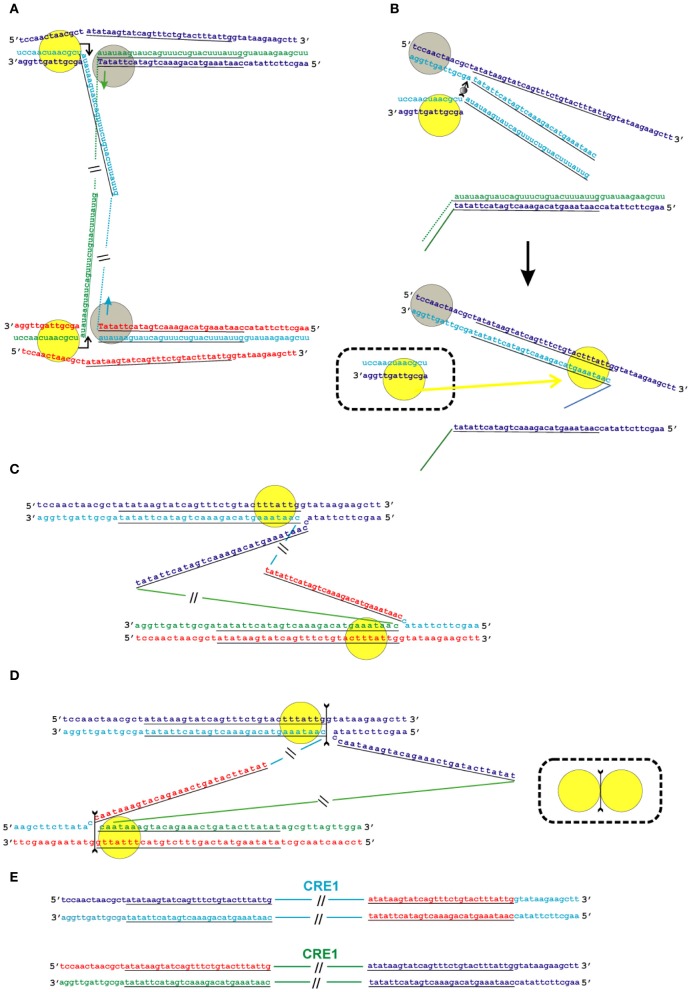
**Model of the transposition of the R2 group of non-LTR retrotransposons for which the endonuclease makes the second DNA nick downstream of the first one.** Sequences correspond to target sites of the *Crithidia fasciculata* CRE1 non-LTR retrotransposon localized within a miniexon. The sequences that correspond to the duplications of the target site are underlined. A schematic presentation of two retrotransposon proteins bound to the RNA co-transcript that contains both the retrotransposon sequence and sequences that flank the target site is not shown. **(A)** Proteins, while bound to the RNAs, bind DNA target sites that are located on the homologous chromosomes. The proteins contribute to the melting of the DNA strands, and RNA/DNA duplexes (R-loops) are formed. Dotted lines correspond to the sequences of the mobile element RNAs. The protein bound close to the 5′-end of the RNA (yellow circle) makes the first ssDNA nick (depicted by a bent arrow). Target primed reverse transcription is started by the protein bound close to the 3′-end of the RNA (gray circles). The nucleotides that are donors of the hydroxyl group are depicted in upper-case letters. **(B)** Here, only events that occur within a single chromosome are shown. When synthesis of the retrotransposon cDNA (solid line) is complete, the protein (gray circles) rests against the RNA/DNA hybrid. Subsequently, this protein jumps to the corresponding free single DNA strand (the jump is depicted by an arrow with a small gray circle) and continues synthesis of the complementary DNA strand. The protein marked by a yellow circle moves (yellow arrow) from the end of the RNA/DNA hybrid to the newly synthesized dsDNA. It is most likely that the RNA in the RNA/DNA hybrids is digested by endogenous host RNase H. The bold black arrow shows the change in conformation of the DNA strands at the 5′-end of the target sites. The sequence that corresponds to the target site duplication anneals to the complementary strand and the protein (yellow circle) moves (yellow arrow) to the end of the dsDNA. **(C)** As a result of the processes described above, a typical Holliday junction structure is formed, with two proteins (yellow circles) bound to the target sites that are located in the homologous chromosomes. **(D)** Owing to the spatial complexity of the structures shown in the figure, two proteins (yellow circles) are shown separately; however, according to the model, they form a dimer that shows Holliday junction-resolving activity. The second cut is shown by an arrow with two feathers. **(E)** The copies of the retrotransposon that are integrated into the homologous chromosomes are shown. The host DNA polymerase completes the synthesis of the second DNA strand that corresponds to the mobile element.

Given that the position of the second nick in relation to that of the first nick varies in different groups of TE, we propose two possible schemes for the transposition process: the first one applies to transpositions with deletion of the target site (as an example, the target site sequence of *B. mori* R2 is used; Figure 5), and the second one applies to transpositions with duplication of the target site (as an example, the target site sequence of *Crithidia fasciculata* CRE is used; Figure 6). We believe that the mechanism of transposition of TEs that make a blunt EN cut can be deduced easily from the two proposed schemes. The main innovation of the proposed models is the assumption that the transposition of R2 group retrotransposons occurs through the formation of Holliday junction structures and their subsequent resolution.

It is clear that, for successful reverse transcription, which is necessary for retrotransposition, both an RNA template and the protein encoded by the retrotransposon are required. In this article, we will not discuss the translation of TEs, although it should be noted that, in the case of R2 retrotransposons, to date, many unresolved issues remain. Probably, for correct translation of the retrotransposon proteins HDV-like ribozyme activity is used by R2 and similar retroelements to efficiently self-cleave the cotranscribed RNA moiety from cotranscript (Eickbush and Eickbush, 2010; Moss et al., 2011; Ruminski et al., 2011).

Given that we suggest that the retrotransposons considered in the present study are transcribed together with the DNA regions that surround their target sites, it would be logical to assume that these co-transcripts serve as templates for reverse transcription. Indeed, the presence of stretches of target site RNA flanking the retrotransposon RNA can greatly facilitate the process of site-specific transposition.

As indicated above, the interaction of R2 proteins with both the 3′- and 5′-ends of the retrotransposon RNA has been shown in experiments *in vitro*. We suggest that transposition starts with the interaction of the RNP complex with the target site DNA in such a way that one end of the RNA forms an RNA–DNA duplex in the integration site that is located on one chromosome, whereas the other end of the RNA forms a duplex in the integration site located on the homologous chromosome (Figures 5A,B and 6A). We assume that duplexes are formed by DNA encircling the integration site and RNA that is homologous to the flanking sequences in the complex co-transcripts described above. To show interacting homologous chromosomes in Figures 5, 6, we adopted the generally accepted model of DNA double-stranded break repair by homologous recombination (Dudás and Chovanec, 2004). According to our model, while the RNP complex interacts with the target site, the R-loop is formed and the protein that is bound closely to the 5′-end of the RNA (yellow circle in Figures 5, 6) makes the first ssDNA nick (depicted by a bent arrow in Figures 5B, 6A). Similar, instead of homologous chromosomes, the sister chromatids could be used.

In the next stage, the TPRT is initiated by a protein that is bound closely to the 3′-end of the RNA (gray circles in Figures 5, 6). The nucleotides that are donors of the 3′-hydroxyl group are depicted as capital letters in Figures 5C, 6A. This stage of transposition of retroelements is the best studied and is present in all models of the transposition of non-LTR retrotransposons that have been proposed to date (Fujimoto et al., 2004; Eickbush and Jamburuthugoda, 2008; Han, 2010).

When synthesis of the retrotransposon cDNA is complete, the protein that carries out the reverse transcription rests against the RNA/DNA hybrid. Subsequently, this protein jumps to the corresponding free single DNA strand (the jump is depicted by an arrow with a small gray circle) and continues synthesis of the complementary DNA strand (Figures 5D, 6B). The possibility of such a jump was shown in experiments *in vitro* that confirmed that R2 reverse transcriptase has high processivity on DNA templates (Bibillo and Eickbush, 2004).

During the next step, RNA is removed from the RNA/DNA hybrid, probably due to the activity of endogenous RNase H. It is known that, in eukaryotic cells, a certain level of RNase H-like activity is maintained (Cerritelli and Crouch, 2009).

If transposition leads to the deletion of a few nucleotides in the target site, the flanking sequences of the co-transcript do not contain these nucleotides, whereas the native integration site does [Figure 5B, two nucleotides marked in black (gg)]. If transposition leads to the duplication of a few nucleotides in the target site, the co-transcript contains these nucleotides on both ends, whereas the native integration site contains only one copy of these nucleotides (the duplicated sequences are underlined in Figure 6). The next step in our model of transposition is slightly different for these two cases. In the second case, cDNA that corresponds to the duplication anneals to the complementary DNA strand (Figure 6B).

On the basis of the experimental evidence that R2 EN is highly sequence-specific to its target site on double-stranded DNA and can cleave ssDNA that extends from the ends of the dsDNA region (Kurzynska-Kokorniak et al., 2007), we propose that the protein that makes the first DNA nick (yellow circle in Figures 5, 6) will move to the end of the dsDNA helix after a complementary DNA strand is synthesized (this move is shown by a yellow arrow in Figures 5D, 6B).

As a result of the processes described above, the typical Holliday junction structure is formed, with two proteins (yellow circles in Figures 5E, 6C) bound to the target sites that are located on the two homologous chromosomes. One of the homologous chromosomes turns around, and the retrotransposon proteins form a dimer (Figures 5F, 6D). The ability of R2 proteins to form dimers has been shown previously. In the present paper, we show the similarity of the R2 protein to Holliday junction-resolving enzymes, which are also homodimers. According to our model, the R2 proteins in dimeric form possess Holliday junction-resolving activity and make the second nick (shown by an arrow with two feathers in Figures 5F, 6D). The host DNA polymerase completes the synthesis of the second strand that corresponds to the mobile element sequence. Owing to the 5′-3′ exonuclease activity of this enzyme, the non-complementary nucleotides (gg) are removed. The copies of the retrotransposon that are integrated into the homologous chromosomes are shown in Figures 5G, 6E.

The scheme of retrotransposon transposition presented above involves the simultaneous participation of two molecules of RNA and, therefore, the simultaneous integration of two copies of the mobile element into two complementary sites on homologous chromosomes. This retains the integrity of the two chromosomes, and the Holliday structures that are formed as described above have the typical architecture. However, the basic scheme outlined above is also applicable for cases in which transposition involves a single molecule of retrotransposon RNA and, consequently, integration occurs into a target site on only one of the homologous chromosomes. Clearly, in this case, the formation of the two initial single-stranded nicks on homologous chromosomes must involve two proteins that form a complex with the RNA retrotransposon: the protein bound to the 5′-end of the RNA makes a single-stranded nick on one chromosome, and the protein bound to the 3′-end of the RNA makes a single-stranded nick on the homologous chromosome. After cDNA synthesis is complete, a structure similar to the Holliday junction structure, with a single-stranded nick, is formed. Resolution of the Holliday junction results in a double-stranded nick on one of the homologous chromosomes, that in which no integration of a TE occurs. To restore the integrity of this chromosome, the DNA repair machinery is required.

## Concluding remarks

At present, several models exist for transposition of non-LTR retrotransposons in the R2 group. All proposed models suggest that R2 EN first nicks one strand of the chromosomal target site. The 3′-hydroxyl group that is released by this nick is then used as the primer for the retrotransposon RT to prime cDNA strand synthesis. *In vitro* experiments showed that R2 RT efficiently uses cDNA as a template for completing retrotransposon integration (Kurzynska-Kokorniak et al., 2007). To explain the *in vivo* data homologous or non-homologous recombination between the 3′-end of the cDNA and the target site sequences before second-strand synthesis of the retrotransposon was suggested. In this case, the second DNA strand could be completed by host DNA repair machinery (Fujimoto et al., 2004). Finally, to explain transposition of the fungal MoTeR elements into telomere repeats, annealing between the 3′-end of the top strand of the nicked telomeric DNA and the short RNA fragment predicted to occur at or near the 3′-end of the MoTeR transcript was proposed. Note that, unlike other models that assume that the first single-strand nick is on the “−” strand of the DNA target site, the fungal MoTeR transposition model suggests that the first nick is on the “+” strand of the telomere repeat (Starnes et al., 2012).

We do not consider our model to be opposed to previously proposed retrotransposon transposition models. Our model was based on experimental results previously used by others to model possible transposition mechanisms. We propose that our model be considered as a special case, appropriate for TEs with site-specific integration that can be transcribed with target sequences and possess EN domains that are similar to Holliday-junction resolvases. Our hypothesis best explains the site specificity of transpositions. Rather than a single universal mechanism of transposition, different, non-mutually exclusive versions of the transposition machinery might function in different eukaryotic organisms and for different non-LTR retrotransposons.

The presence of fused RNA transcripts corresponding to TEs and sequences that surround their target sites enables the formation of R-loops; these can alleviate the process of transposition and promote site specificity. The four-way junction in our model occurs only if the integrated copies of retrotransposons are transcribed together with their target sequences and R-loops are formed at homologous chromosomes or sister chromatids. According to our model, four-way junction formation requires only RNA annealing and subsequent cDNA synthesis.

At certain stages of replication cycle, retrotransposons (as well as many viruses) use enzymes encoded by host genomes in addition to enzymes encoded by their own genomes. Different models of non-LTR retrotransposon transposition have been proposed to date that suggest participation of host enzymes for filling single-stranded gaps, removing non-homologous flaps, DNA strand ligation (Eickbush and Jamburuthugoda, 2008; Han, 2010), and/or homologous recombination during integration of the 5′-end of retrotransposon DNA (Fujimoto et al., 2004). The Holliday structure resolution that is crucial for transposition in our model also requires participation of host enzymes, such as helicases and/or topoisomerases.

Of note, our model for retrotransposon transposition cannot explain the very first integration of a retrotransposon into a genome. However, this event occurred millions of years ago in an ancestral form of modern organisms. Previously described models are appropriate to explain how this first integration happened. For example, the EN domain of the *B. mori* R2 retrotransposon makes both the first and second single-stranded breaks of target sequences (Kurzynska-Kokorniak et al., 2007) necessary for the initial integration. Since that time, the transposition mechanism could have undergone evolutionary changes to adapt to new integration sites, in particular, regions that are transcribed together with the integrated copies of TEs. Probably, the first retrotransposons were not integrated site specifically into actively transcribed regions; these integration sites came later. The ability of the EN domains to resolve four-way junctions would be useful at that time, to simplify integration and promote site specificity. During evolutionary changes in TEs, successive shifts in target site preferences might have occurred. This could be followed by adaptive changes in the structural organization of mobile element proteins. Together, these events might complicate both the classification of TEs, and attempts to relate the specific transposition mechanisms to particular phylogenetic clades of TEs.

Finally, the key provisions of our model can be tested experimentally. The ability of EN domains to resolve the four-way junctions can be tested *in vitro*. A four-way DNA junction substrate with a core sequence corresponding to Figures 5, 6 and purified proteins from the ORF of non-LTR retrotransposons could be used. A similar approach was used to analyze Hjc enzymatic activity (Kvaratskhelia et al., 2000). The role of target sequences co-transcribed with the retrotransposons in transposition could be assessed by an experimental strategy described in Roy et al. (2010). The protocol allows analysis of the competition between an RNA transcript and a non-template DNA strand during the R-loop formation *in vitro*.

### Conflict of interest statement

The authors declare that the research was conducted in the absence of any commercial or financial relationships that could be construed as a potential conflict of interest.
